# Introducing the PLOS ONE Collection on the neuroscience of reward and decision making

**DOI:** 10.1371/journal.pone.0240505

**Published:** 2020-10-08

**Authors:** Stephanie M. Groman, Satoshi Ikemoto, Matthew Rushworth, Jane R. Taylor, Robert Whelan

**Affiliations:** 1 Department of Psychiatry, Yale University, New Haven, CT, United States of America; 2 National Institute on Drug Abuse, National Institutes of Health, Bethesda, MD, United States of America; 3 Department of Experimental Psychology, University of Oxford, Oxford, United Kingdom; 4 School of Psychology, Trinity College Dublin, Dublin, Ireland; Texas A&M University, UNITED STATES

## Abstract

The survival of an organism depends on the ability to make adaptive decisions to achieve the needs of the organism: where to get food, who to mate with, and how to evade predators. Decision-making is a term used to describe a collection of behavioral and/or computational functions that guide the selection of an option amongst a set of alternatives. Some of these functions may include calculating the costs and benefits of a particular action, evaluating differences in value of each of the alternative outcomes and the likelihood of receiving a particular outcome, using past experiences to generate predictions or expectations about action-outcome associations, and/or integration of past experiences to make novel inferences that can be used in new environments. There is considerable interest in understanding the neurobiological mechanisms that mediate these decision-making functions and recent advances in behavioral approaches, neuroscience techniques, and neuroimaging measures have begun to develop mechanistic links between biology, reward, and decision making. This multidisciplinary work holds great promise for elucidating the biological mechanisms mediating decision-making deficits in normal and abnormal states. The multidisciplinary studies included in this Collection provide new insights into the neuroscience of decision making and reward.

## Introduction

Decision making describes the process by which an organism selects an option amongst a set of alternatives that are expected to result in different outcomes [1]. Selection of a particular action may be based on evaluations that include calculating the desirability of each alternative outcome, determining how likely it is that a particular alternative is going to result in each outcome, or weighing the costs and/or benefits of choosing one alternative over the others [2–4]. The degree to which these evaluations can account for differences in choice behavior and how these processes are performed can vary between individuals and, importantly, differ between normal and abnormal states [5, 6]. Understanding the neurobiological mechanisms that mediate select aspects of reward and decision-making functions, therefore, could provide critical insights into the neural systems that are altered in mental illness [7–9].

There have been significant advancements in the neuroscience techniques capable of probing the cellular and systems-level mechanisms of reward and decision making have occurred in the past several years. We are now able to manipulate specific neural populations and circuits [10], alter expression of gene(s) with remarkable temporal precision [11], record single neuron or population activity simultaneously across various brain regions [12, 13], and obtain high resolution *in vivo* measures of the brain [14–16] that can be linked to precise behavioral events. Integrating these tools with sophisticated behavioral and computational analyses in humans and animals could help to develop mechanistic bridges between biology and complex decision-making processes and improve our neuroscientific understanding of reward and decision making [17]. Furthermore, this integrative approach holds great promise for dissociating the neurobiological mechanisms that underlie decision making across disease states.

The articles in this Collection highlight the diversity of research that is being conducted to advance our understanding of the neuroscience of reward and decision making in humans and non-human animals. The studies included this Collection use sophisticated behavioral approaches in both humans and non-human animals, circuit-based approaches to characterize and manipulate pathway specific projections, transgenic rodents to ablate select cells types, and biologically based neural network models to link genetic variability to behavior. These articles are grouped into three main themes: 1) human-based investigations into reward and decision making, 2) non-human animal investigations into reward and decision making, and 3) investigations into aging and abnormal reward and decision making. We provide a brief summary of each paper followed by a discussion of the research presented in this Collection.

### Theme 1: Human-based investigations into reward and decision making

Reward-guided learning and decision making is influenced by violations of expectations and uncertainty in value–if the actions we perform are incongruent with the value of the outcomes we receive or the timing in which we expect these outcomes to occur, then the representation of the action-outcome association needs to be updated and we may shift our strategy from one of exploitation to exploration. Individual differences in how these select processes occur may be related to variation in the activity of select brain regions or sensorimotor noise that exists within the system. The three studies presented under *Theme 1*: *Human-based investigations into reward and decision making* examine how local and global violations of expectations covary with electroencepahalogram (EEG) recordings of brain activity, value uncertainty relates to fMRI-based changes in blood-oxygenated levels, and how exploration can be quantified in reward-based motor learning to estimate sensorimotor noise in humans.

#### Brain activity changes in response to local and global surprise

Several studies have been conducted with human participants and a variety of methods have been used to record neural activity while they process carefully manipulated variations in uncertainty. **Kluger and colleagues** [18] recorded the EEG from scalp electrodes while people responded to particular events of importance that occurred within a series of background events. We know that some of the largest signals in the EEG are very sensitive to surprising events and the need to update future expectations about what might happen next [19–21]. In the new study that Kluger and colleagues report, human participants were asked to watch a series of digits being presented. The participants responded to consecutive digit strings as opposed to non-consecutive digit strings. Expectations about the length of the consecutive strings, however, were manipulated in two ways. First, local cues, presented at the time of the string itself, predicted if the sequence was going to be longer than usual but these cues were only probabilistic. Aspects of the EEG, decodable with a multivariate analysis approach and present at the time of the P3b evoked potential response (ERP; time-locked EEG), distinguished whenever the cues indicated a need to update the usual expectations about what was going to happen next. As well as looking at whether the EEG reflected the occurrence of an individual event requiring the updating of expectations, Kluger and colleagues examined whether it was sensitive to the rate at which surprising events occurred. To do this they varied the rate of surprising events throughout the block of trials and found that the EEG in the P3b time period also reflected the rate of surprising events. Kluger and colleagues argue that we should think of activity in the P3b period as not just reflecting *local* instances of a need to update our model of the world but of global shifts in how likely such needs may be. In other words, together these results suggest that the EEG reflects both local indicators that a surprising event is likely to happen now and the global rate at which they are likely to happen. In addition to these responses to cues that are predictive of surprising events, Kluger and colleagues also recorded neural activity in response to the surprising events themselves. There are two ways in which an event is surprising in this task. On some occasions the longer sequence that the predictive cues had foretold did not then actually happen and, on some occasions, when a normal sequence length was predicted, the sequence actually turned out to be longer than expected. The EEG-recorded N400 ERP, a mismatch signal, was greater whenever the events ran counter to these local expectations. While the results are intriguing, integrating an understanding of such well known EEG signals with an understanding of the prediction error and surprise signals that can be recorded from specific anatomical structures remains elusive. While comparing different levels of surprise can be informative, it might also be interesting to use more computationally-inspired approaches to track the evolution of the participants’ expectations across the session in order to obtain parametrically varying indices of surprise.

#### Value uncertainty co-varies with BOLD responses in the vmPFC

A different approach to the measurement of uncertainty was taken by **Shapiro and Grafton** [22]. Like Kluger and colleagues, Shapiro and Grafton looked at human behavior but instead of EEG, they used functional magnetic resonance imaging (fMRI) to record brain activity. Rather than looking at brain responses to surprising visual events, however, Shapiro and Grafton were interested in uncertainty in the estimate of the value of a choice. A choice might be good or bad. It might have a high value or a low value. However, in addition, a person’s certainty about that estimate may also vary. Two choices might be estimated to have the same value but in the case of one choice, the value estimate may be known with certainty but in the other case there might be uncertainty about the estimate. We know from human functional magnetic resonance imaging (fMRI) studies that activity in the ventromedial prefrontal cortex (vmPFC) often reflects the value a person assigns to a choice that they are considering taking [23–25] but in addition a separate part of the variance in the blood oxygen level dependent (BOLD) signal reflects uncertainty about the value estimate [26, 27]. Shapiro and Grafton used a new behavioral task for human subjects based on one previously used with macaques [28]. An elegant feature of the task is that each choice has good and bad elements. In the case of the human participants studied here, each choice’s value comprises a good component reflecting a monetary reward and a bad component reflecting an electrical shock. As the good and bad components are parametrically varied, participants become more or less likely to take a choice. Shapiro and Grafton carefully estimated the values of the choices and the uncertainties in these estimates. Not only were they able to demonstrate that both of these important determinants of choice selection were represented in vmPFC but they were able to show that value-related activity emerges first as participants contemplate a possible choice and then uncertainty-related activity comes next as participants decide whether or not to take the choice. While the temporal separation between the two signals is striking, its implications for understanding the precise nature of vmPFC’s contribution to decision making remain unclear.

#### Sensorimotor noise can be used to estimate exploration in reward-based motor learning

Aspects of motor learning may also involve adapting to uncertain environments. Variability in movements may be the result of exploration and sensorimotor noise, such that one might adjust (or explore) postural shifts in response to movement of shaky train to stop falling, yet given that motor movements are inherently noisy (sensory motor noise) variations on posture might be cause by a combination of these factors. Previous studies into reward-based motor learning have shown a higher variability in motor output following non-rewarded movements than following rewarded movements. However, exploration is difficult to measure because variability consists of multiple sources of sensorimotor noise that include planning noise, execution noise and perceptual noise. **van Mastrigt and colleagues** [29] investigate ways of quantifying exploration in reward-based motor learning by systematically manipulating variability due to sensorimotor noise. A target directed, reward-based weight-shift task was employed where multiple different baselines could be used to calculate variability due to exploration as opposed to that due to sensorimotor noise, where participants performed baseline blocks without feedback, and the several blocks of trials that alternated with and without feedback. Because variability was greater following non-rewarded trials than rewarded trials, these results suggest that estimates of performance based on trial-to-trial changes following reward provided a better baseline than trials in a no-feedback block during test sessions, and these authors suggest that sensorimotor noise may increase when exploring because reward is more uncertain when exploring. Interestingly, the task was not influenced by motivation or learning factors per se during the test sessions. These data suggest that the reward-based weight-shifting task successfully induced exploration and that exploration can best be quantified using sensorimotor noise estimated from trial-to-trial changes following rewarded trials. Both the planning and execution of movements are inherently noisy and how individuals integrate exploration into the variability of movement output may be a key component of motor learning that can be studied with quantitative estimates of variability. Studies that use this target-directed weight-shifting task with stochastic reward feedback to precisely quantify exploration in reward-based motor learning may lead to a better understanding of how sensorimotor noise and exploration represent sources of variability in human motor performance in uncertain or dynamic environments.

### Theme 2: Non-human animal investigations into reward and decision making

Variation in reward and decision-making functions may be governed by select genes, neural circuits, and cell-types within the brain. Studies in non-human animals have the ability to probe select biological mechanisms with greater specificity and resolution than that typically afforded in studies using human subjects. Moreover, causal manipulations can be conducted in non-human animals to test the correlative results obtained in human subjects. The studies presented under the theme of “Non-human animal investigations into reward and decision making” in this Collection represent the integrative and innovative work that is being conducted in non-human animals to identify the genetic, neurobiological, and circuit-level mechanisms of reward-guided behavior. The four studies included under this theme investigate the role of a novel immediate early gene in reward-guided foraging behavior in the bee, the necessity of dopamine signaling in optogenetically-mediated self-stimulation of the lateral hypothalamic medial forebrain bundle, the role of select lateral hypothalamic sub-regions in self-stimulation and feeding behavior, and the involvement of striatal patches in the formation of habitual behaviors.

#### The immediate early gene kakusei is associated with reward-related behavior in bees

Foraging is a highly complex social behavior that involves aspects of learning, motivation, and communication, and studies aimed at understanding the cellular and molecular underpinnings of foraging are likely to reveal systematic and dynamic components of goal-directed behaviors. Although behavioral studies of foraging in bees is an established model system, its molecular and cellular mechanisms have only recently been studied. The manuscript by **Singh and colleagues** [30] focused on the expression of the kakusei gene–a recently discovered immediate early gene (IEG) that was first identified in Kenyon cells of foraging bees. IEGs are rapidly induced in brain in response to environmental stimuli and are known to reflect synaptic activity and to play a critical role in learning and memory processes across many species. The IEGs early growth response 1 (egr-1) and nuclear hormone receptor 38 (hr38) and their corresponding partners have been reported to be involved in aspects of learning and memory in bees and Drosophila, but the kakusei gene had not been studied within the context of learning and memory, and foraging specifically. Several of these other well studied IEGs, known to associate with patterns of neuronal activity in foraging bees, were also examined and compared with measures of kakusei. Using a daily foraging paradigm, only the kakusei gene was found to be associated with a transient and prolonged upregulation that occurred during reward foraging and a short period of overexpression during unrewarded foraging. The presence of food reward was found to be essential for the increased expression and sustained higher kakusei levels during foraging, which establishes kakusei as a novel IEG foraging-regulated gene that may be uniquely related to reward-related motivation. These data also suggested a possible role for kakusei in learning and specifically for the memory of the location of food rewards and hive location during foraging. Future studies aimed at confirmation of these putative learning and memory functions of kakusei and other IEG, identification of downstream signaling pathways, and their involvement in precise components of social interaction and communication among the foraging honey bee as a model system will help elucidate complex and dynamic social and decision-making processes across species. The complexity of foraging and relevance to decision-making processes likely make it applicable to aspects of human learning and memory processes that also involve social communication.

#### The lateral hypothalamic medial forebrain bundle and dopamine projections are parallel limbs of brain-reward circuitry

The research on the brain reward system arose out of the discovery that rats learn to self-administer electrical stimulation at discrete brain regions, behavioral phenomena referred to as intracranial self-stimulation (ICSS). This research led to the lateral hypothalamic medial forebrain bundle (LH-MFB) as one of the most effective areas supporting ICSS and dopamine as a key molecule of the reward system. Contrary to the established view that the activation of dopamine neurons is an obligatory component for ICSS reinforced by LH-MFB stimulation, Shizgal’s group recently obtained data that are not consistent with this view. They found that dopamine manipulations can significantly alter the opportunity cost, but not the strength, of reward-seeking responding reinforced by LH-MFB stimulation. In the present study, **Trujillo-Pisanty et al.** [31] examined the effects of dopamine transporter blockade on reward-seeking responding reinforced by optogenetic stimulation of ventral tegmental dopamine neurons, while varying the reward strength and opportunity cost of the stimulation. The selective dopamine transporter blocker GBR-12909 affected both strength and cost measures of dopamine neuron stimulation. Given that the same transporter blocker only affects the cost, but not the strength, measure with LH-MFB ICSS, the authors proposed a parallel model in which LH-MFB stimulation and dopamine neuron stimulation activate two separate pathways that ultimately converge on a common pathway. This study raises a fundamental question on the organization of the brain reward system.

#### Self-stimulation of the tuberal lateral hypothalamus supports stimulus-bound feeding

The lateral hypothalamic area (LH) has been known to support not only ICSS, but also induce stimulus-bound behavior in which electrical stimulation applied at the LH induces feeding, gnawing, etc. Because electrical stimulation affects various elements of neurons, including the fibers of passage, it is not known how much of stimulation-induced behavior is contributed by the neurons at the stimulation site. To get around this issue, **Urstadt and Berridge** [32] used optogenetic procedures to selectively stimulate local neurons within the LH of rats. They compared the sub-areas of the LH, to determine the most effective sub-areas for induced feeding and self-stimulation (i.e. positive and negative incentive effects). They found that the tuberal LH most effectively supported stimulus-bound feeding. Interestingly, repeated stimulation of this region resulted in increased effectiveness in self-stimulation. The lateral preoptic area and posterior LH did not readily induce feeding or self-stimulation. These findings indicate that subregions of the LH are uniquely involved in feeding and reinforcement.

#### Ablation of striatal patches disrupts the formation of habitual behaviors

The dorsal striatum plays a critical role in development of habitual behavior. Specifically, the lateral part of the dorsal striatum is thought to be critical in habit formation, while the medial part is important for producing goal-directed behavior. In addition to this medial-lateral function distinction, the dorsal striatum consists of two distinct intermingled zones: the patch and matrix. It is unclear what the function of this patch/matrix organization is. **Nadel and colleagues** [33] examined the role of patches in habits, using the transgenic mice that expresses Cre recombinase in neurons containing Sepw1 NP67, which is expressed preferentially in patches. While the lesions of Cre neurons with caspase 3 did not disrupt the acquisition of instrumental responding for sucrose reward, the lesions increased goal-directed strategies and decreased stability in performance levels across sessions compared to sham lesions. These effects of the lesions support the view that without patches, operant behavior is kept regulated by goal-directed processes and not readily transformed into fixed, habitual behavior. Therefore, these results suggest that striatal patches play an important role in habitual behaviors.

### Theme 3: Investigations into aging and abnormal reward and decision making

Disruptions in the neural mechanisms mediating reward and decision making may explain how differences in choice behavior emerge in normal and clinical populations. The manuscripts under the theme of “Investigations into aging and abnormal reward and decision making” in this Collection describe work that is being done to understand how external (e.g., aging, alcohol exposure) and internal (e.g., genetic variability) factors impact reward and decision making. The three papers described under this theme examine how feedback learning may differ between younger and older adults, alcohol-induced changes in connectivity of the globus pallidus externus, and how mutations in the TOR1A gene can alter neural networks that are relevant to risky decision-making.

#### Feedback-based learning is reduced in older adults compared to younger adults

Previous research has shown that older adults may have a reduced capacity to learn from feedback, possibly due to an age-related reduction in the dopaminergic reinforcement learning signal. In contrast, it is thought that processing of emotional stimuli is relatively unaffected in older adults. **Ferdinand and Hilz** [34] examined if the use of emotional, rather than abstract, feedback could help attenuate deficits in instrumental learning in older adults. They used a combination of behavioral and EEG measures: the latter allowed the non-invasive measurement of brain activity with very high temporal precision. Participants–in younger and older adult groups–engaged in a probabilistic learning task that had emotional and non-emotional feedback conditions. Ferdinand and Hilz hypothesized that the provision of emotional feedback would result in faster learning and enhanced feedback processing, quantified by larger ERP components, particularly for the older adults. Participants were presented with a cover story: their task was to load objects into either a black or white truck. A ‘superior’ would provide feedback, which was accurate 90% of the time. In the emotional feedback condition, the superiors’ facial expression was either friendly or disgusted. In the non-emotional feedback condition, background color denoted the contingencies. As expected, the younger adults were better at learning the task. However, both behavioral and EEG data showed that the performance of older adults was improved in the emotional condition, a corresponding performance improvement that was not seen in the younger group. Ferdinand and Hilz’s study highlight the importance of considering affective factors in reward-based decision making, and how these factors may change over the course of the lifespan.

#### Alcohol-induced changes in connectivity of the globus pallidus related to patterns of alcohol use

Addiction is a disorder characterized by aberrant decision-making and reward processing. These behaviors are typically studied by comparing heavy alcohol users to lighter users, notably when subjects are not under the influence of alcohol. The basal ganglia, which lie deep inside the brain, play an important role in control over impulsive behavior as part of a larger network involving frontal brain regions [35]. The connectivity among structures within the basal ganglia is well described with respect to inhibiting inappropriate responses. The globus pallidus externus (GPe) belongs to the ‘indirect’ basal ganglia pathway, connecting to the subthalamic nucleus and substantia nigra and to the striatum. A non-human animal model [36] has shown that alcohol decreased neuronal firing rate in the GPe. There is, however, little direct study of functional brain connectivity changes resulting from alcohol consumption in humans, even though disinhibited decision making is perhaps the most well-known acute effect of alcohol. **Fede and colleagues** [37] sought to address this gap by investigating how GPe connectivity changed under the administration of alcohol. Furthermore, they related this connectivity to behaviors relevant to addiction: namely, drinking patterns, trait impulsivity and their interaction. Under functional magnetic resonance imaging, 25 healthy adults were administered alcohol intravenously to a level consistent with binge drinking for a period of 30 mins. Fede et al.’s findings showed that the interaction between alcohol-induced connectivity, personality and drinking patterns is more nuanced than originally thought. For example, participants with a recent history of heavier drinking, relative to lighter drinkers, had greater coupling between the GPe and frontal brain region in the absence of alcohol–a pattern that was reversed in the presence of alcohol. With respect to impulsivity, in the absence of alcohol there was no relationship between impulsivity and connectivity between the GPe and brain areas involved in motor inhibition. However, the GPe had increased connectivity to these areas under alcohol infusion. A notable conclusion from the work of Fede and colleagues is that the relationship between decision-making and reward-related behavior substance may be different under acute intoxication versus non-intoxication. One limitation of Fede et al. is the modest sample size (n = 25), and replication of their results is important, in order to confirm the relatively complicated relationships among brain and behavioral variables. Future research could also examine connectivity among other basal ganglia regions to more fully characterize how connectivity changes under alcohol administration relate to decision making.

#### Disruptions in striatal networks may explain the decision-making impairments associated with the TOR1A mutation

The likelihood of obtaining a desired outcome based on a particular action or choice is not always guaranteed and may be associated with an element of risk or uncertainty. Decision making under risky or uncertain environments may be governed by plasticity-related mechanisms in the cortico-striatal circuitry that are regulated by distinct genetic mechanisms. The *TOR1A* gene is known to play a critical role in mediating long-term potentiation and long-term depression in cortico-striatal synapses and previous work has reported that patients with the *TOR1A* mutation are more likely to make risky choices compared to controls. Different theories have been proposed for how variation in plasticity-related mechanisms mediated by the TOR1A gene might lead to these behavioral differences, but some of these explanations are incongruent with *in vitro* measurements collected in rodents with the humanized TOR1A mutant gene. Here, **Gilbertson and colleagues** [38] sought to reconcile the experimental observation of increased risky choice behavior in patients with the *TOR1A* mutation and reports of excess cortico-striatal LTP and diminished LTD in rodent genetic models. Using a neural network model of the basal ganglia, Gilbertson et al. were able to simulate choices that were statistically identical to those observed in patients with the *TOR1A* mutation, but not when the pattern of cortico-striatal abnormalities identified in the *TOR1A* rodents were incorporated (e.g., increased long-term potentiation in both direct and indirect medium spiny neurons (MSNs)). Rather, risky choice behavior was recapitulated when increased cortico-striatal long-term potentiation in direct MSNs was combined with increased long-term depression in indirect MSNs. These findings suggest that the cortico-striatal plasticity abnormalities observed in the TOR1A rodent model may differ from those that lead to the risky choice behavior in humans with the TOR1A mutation and provide new insights into the neurobiological mechanisms underlying risky decision making. Moreover, the study by Gilbertson et al. provides a demonstration of how sophisticated reinforcement-learning algorithms that incorporate biological plausible mechanisms can be used to evaluate specific hypotheses and provide neural insights into abnormal decision making.

## Conclusions

The studies included in this Collection provide new insights into the neuroscience of reward and decision making in both humans and non-human animals. The diversity and breadth of the work that is being done in this field–including investigations in humans and non-human subjects and across different levels of analyses (e.g., genes, proteins, circuits, and neural activity)–are well represented in this Collection of papers. For example, the papers discussed under the theme of “Human-based investigations in reward and decision making” demonstrate how surprise, uncertainty, and sensorimotor processes can modulate reward-guided behaviors and are linked to select changes in brain activation patterns. The papers discussed under the theme of “Non-human animal investigations into reward and decision making” identify new gene correlates of foraging behavior, use optogenetic approaches to identify brain regions involved in specific reward-mediated processes, and transgenic approaches to identify how select compartments in brain regions control reward-guided behavior. Finally, the papers discussed under the theme of “Investigations into aging and abnormal reward and decision making” demonstrate the effects of aging on feedback-based decision making, impact of alcohol on neural connectivity measures, and how neural network models can be used to understand the role of genetic variation in decision making.

A major challenge for the field–as evidenced by the diversity of research contained within this Collection of papers–is understanding how the findings from individual studies can be integrated to develop a cohesive framework for elucidating how the brain processes rewards and makes decisions. For example, how might the parallel limbs of brain-reward circuitry proposed by Trujillo-Pisanty et al. [31] modulate changes in prefrontal activity that occur in response to surprise and uncertainty, as reported by Kluger et al. [18] and Shapiro and Grafton [22]? Could risk for developing habitual behaviors be the result of enhanced activity of striatal patches (e.g., striosomes; [33]) that, subsequently, alters the connectivity of the globus pallidus externus in response to psychotropic drugs as was observed by Fede et al. [37]? And how might differences in the activity of specific cells types and circuitry that mediate reward and decision-making [32] be the result of variability in the sequence or expression of particular genes [30, 38] or the result of natural processes such as aging [34]? We suggest, therefore, that the strength of this Collection–which contains novel results in both human and non-human animal subjects at different levels of analyses–is in the use of these studies to generate mechanistic hypotheses of reward and decision making for subsequent investigations.

In summary, this collection of work highlights the utility of an interdisciplinary approach for linking biology with behavior and generating translational bridges between results collected in human and non-human animals that will undoubtedly improve our neuroscientific knowledge of reward and decision making.
